# Supplementary material for: Intraoperative sodium range affects white matter microstructure in neonatal congenital heart disease
Source: Int J Cardiol Congenit Heart Dis. 2026 Mar 5;24:100666. doi: 10.1016/j.ijcchd.2026.100666 (PMC12996680; doi:10.1016/j.ijcchd.2026.100666)

**Supplementary Material**

**Intraoperative sodium range affects white matter microstructure in neonatal congenital heart disease**

Mirthe E.M. van der Meijden^a,b,c^, Kim van Loon^b,d^, Maaike Nijman^a,b,e^, Hanna Talacua^b^, Johannes M.P.J. Breur^b^, Joppe Nijman^b^, Nathalie H.P. Claessens^a,e^, Serena J. Counsell^c^_,_ Manon J.N.L. Benders, MD^a,e^, Alexandra F. Bonthrone^c^

^a^Department of Neonatology, Wilhelmina Children’s Hospital Utrecht, University Medical Center Utrecht, The Netherlands

^b^Center for Congenital Heart Defects Utrecht, Wilhelmina Children’s Hospital, University Medical Center Utrecht, The Netherlands

^c^Research Department of Early Life Imaging, Centre for the Developing Brain, School of Biomedical Engineering and Imaging Sciences, King’s College London, United Kingdom

^d^Department of Pediatric Anesthesiology, Wilhelmina Children’s Hospital Utrecht, University Medical Center Utrecht, The Netherlands

^e^Brain Center Rudolph Magnus, University Medical Center Utrecht, The Netherlands

**Supplementary Figure 1.** White matter regions that show a significant negative relationship between maximum intraoperative sodium range and postoperative AD (red/orange/yellow, p<0.05) in a sensitivity analysis including SCP/DHCA as an additional covariate, overlaid on FA skeleton (blue) and mean FA map (grey). All sagittal images are taken from the right hemisphere.


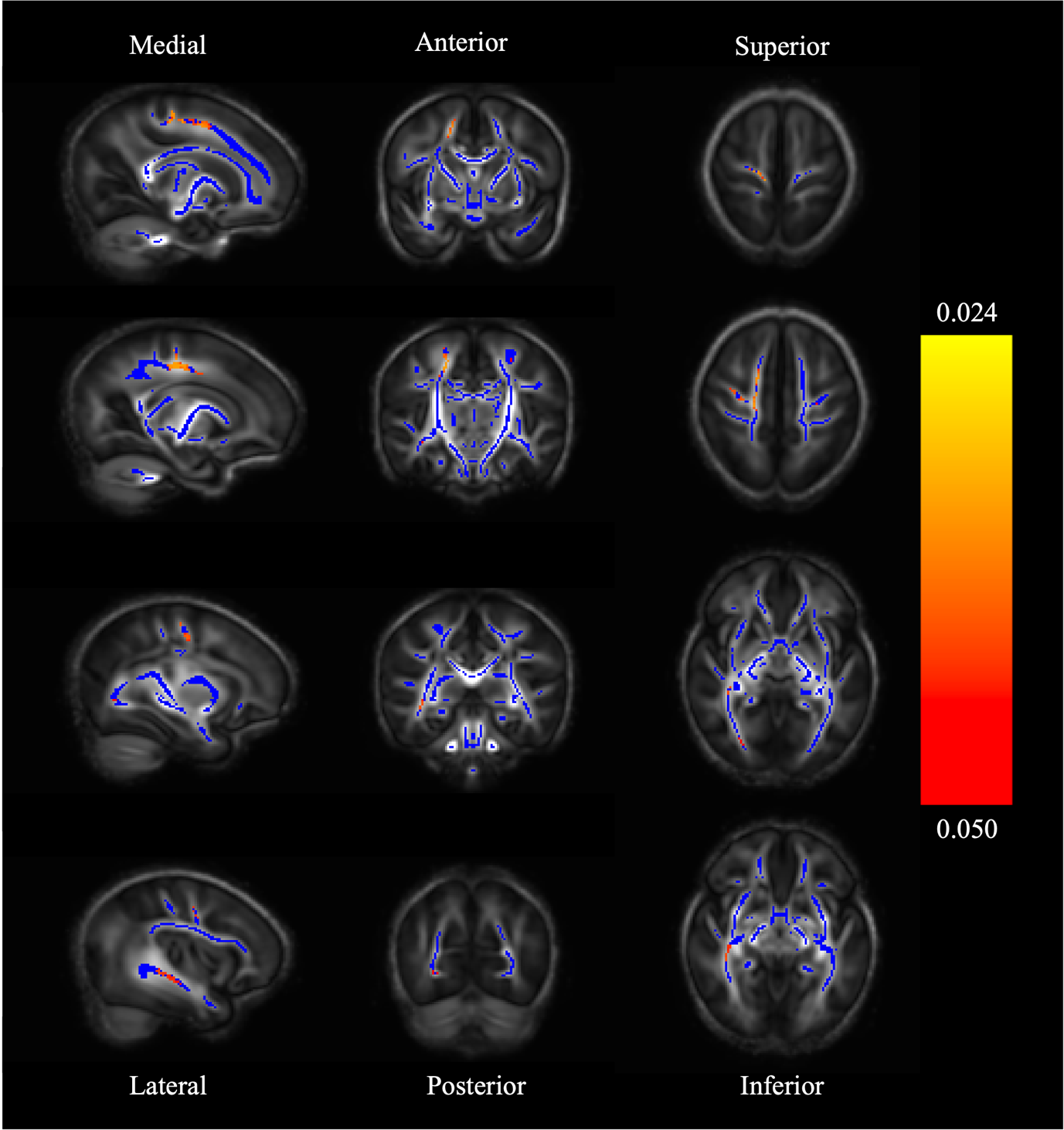


**Supplementary Figure 2.** White matter regions that show a significant negative relationship between maximum intraoperative sodium range and postoperative AD (red/orange/yellow, p<0.05) in a sensitivity analysis including total intensive care length of stay as an additional covariate, overlaid on FA skeleton (blue) and mean FA map (grey). All sagittal images are taken from the right hemisphere.


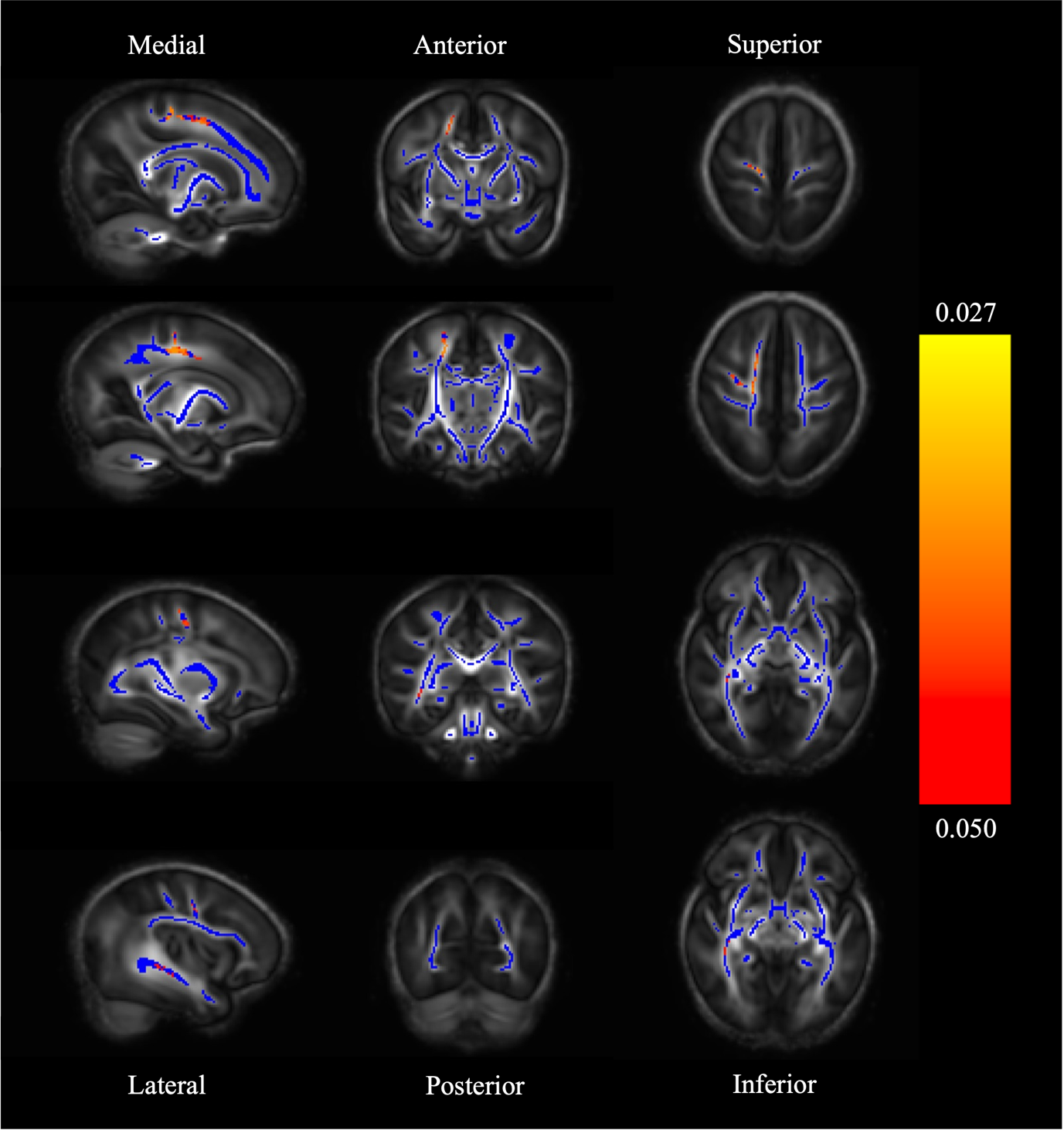


**Supplementary Figure 3.** White matter regions that show a significant negative relationship between maximum intraoperative sodium range and postoperative AD (red/orange/yellow, p<0.05) in a sensitivity analysis including cyanotic heart defect as an additional covariate, overlaid on FA skeleton (blue) and mean FA map (grey). All sagittal images are taken from the right hemisphere.


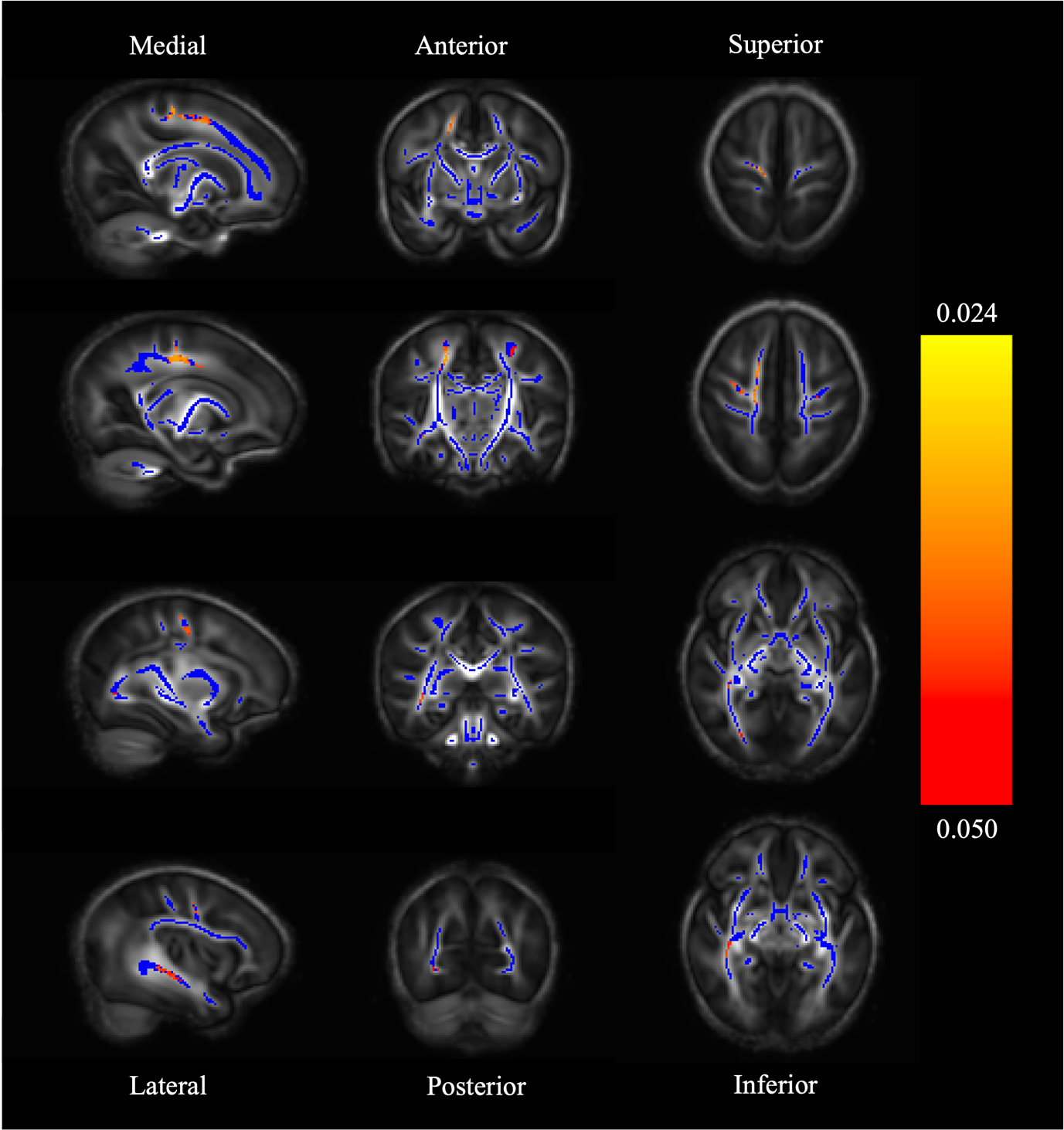

Supplement: Multimedia component 1 [file mmc1.docx]
